# Oral submucous fibrosis stimulates invasion and epithelial‐mesenchymal transition in oral squamous cell carcinoma by activating MMP‐2 and IGF‐IR

**DOI:** 10.1111/jcmm.16929

**Published:** 2021-09-15

**Authors:** Pei‐Ni Chen, Chiao‐Wen Lin, Shun‐Fa Yang, Yu‐Chao Chang

**Affiliations:** ^1^ Clinical Laboratory Chung Shan Medical University Hospital Taichung Taiwan; ^2^ Institute of Medicine Chung Shan Medical University Taichung Taiwan; ^3^ Institute of Oral Sciences Chung Shan Medical University Taichung Taiwan; ^4^ Department of Dentistry Chung Shan Medical University Hospital Taichung Taiwan; ^5^ Department of Medical Research Chung Shan Medical University Hospital Taichung Taiwan; ^6^ School of Dentistry Chung Shan Medical University Taichung Taiwan

**Keywords:** epithelial‐mesenchymal transition, insulin‐like growth factor‐1, invasion, oral cancer, oral submucous fibrosis

## Abstract

Oral submucous fibrosis (OSF) involves a high risk of malignant transformation and has been implicated in oral cancer. Limited studies have been conducted on the role of OSF in relation to the invasive capabilities and epithelial‐mesenchymal transition (EMT) in oral cancer. Herein, we investigated the effects of OSF on the microenvironment of human oral cancer cells. The results showed that the conditioned medium (CM) of fibrotic buccal mucosal fibroblasts (fBMFs) strongly induced the invasion of oral cancer cells and increased the activities of matrix metalloproteinase‐2. OSF significantly induced the EMT in oral cancer cells and downregulated epithelial markers, such as E‐cadherin, but significantly elevated vimentin, fibronectin, N‐cadherin, RhoA, Rac‐1 and FAK. Insulin‐like growth factor‐1 (IGF‐1) was elevated in OSF. The protein levels of the IGF‐1R were upregulated specifically in fBMF CM treatment for oral cancer cells, and the IGFR gene was confirmed by The Cancer Genome Atlas patient transcriptome data. The Kaplan‐Meier curve analysis revealed that patients with oral squamous cell carcinoma and high IGFR expression levels had poorer 5‐year survival than those with low IGFR expression (*p* = 0.004). The fBMF‐stimulated EMT cell model may recapture some of the molecular changes during EMT progression in clinical patients with oral cancer.

## 1 INTRODUCTION

Cancer growth is determined by cell proliferation and relies on the microenvironment of the tumour, which has been recently considered as an attractive target for new anti‐tumour therapies.^1^ The microenvironment of oral squamous cell carcinoma (OSCC) contains many different cell populations, including immune cells, endothelial cells, fibroblasts and non‐cell components of the extracellular matrix.^2^, ^3^ Fibroblasts are the most abundant stromal cells in the tumour microenvironment and can secret a wide spectrum of cytokines and chemokines into the invasive margins of desmoplastic cancers to promote cancer development and activate metastasis.^4^ Recently, collagen cross‐linking and the expression of key enzymes, including lysyl hydroxylase 2 or lysyl oxidase (LOX) and LOX‐like 2 were significantly increased in late‐stage tumours and associated with poor prognosis in patient with OSCC.^5^

Betel quid ingredients initiate deoxyribonucleic acid damage and induce reactive oxygen species production, which can lead to the promotion and progression of oral cancer.^6^ Betel quid chewing is implicated in OSCC and oral submucous fibrosis (OSF), and the prevalence of OSF amongst betel quid chewers ranges from 0.9% to 4.7% in Mainland China.^7^ Betel quid chewing causes the high‐risk alleles and genotypes of transforming growth factor‐β1 (TGF‐β1), matrix metalloproteinases (MMPs) and LOX found in OSF patients may change the function and the expression of corresponding proteins and produce inflammation of fibroblasts and collagen deposition.^8^ Compared with non‐chewers group, betel quid dependency had the higher risk of oral potentially malignant disorders (adjusted odds ratios 8.0–51.3) than participants with non‐dependent betel quid use (0–2 dependency domains; adjusted odds ratio 4.5–5.9) in six Asian populations.^9^

Cancer‐associated fibroblasts (CAFs) play a major regulatory role in matrix remodelling and influence the development of immunosuppression in the tumour microenvironment.^10^ OSF is a chronic inflammatory disease that frequently occurs in buccal mucosa and is a progressive form of the fibrosis of lamina propria and deeper connective tissues.^11^ This condition has been implicated in scar formation, tissue fibrosis and precancerous lesions due to its various mechanisms. Patients with OSF have a high correlation of oral cancer.^12^ In oral cancer, OSF causes predisposition to its development and has tumour‐promoting effects.^13^ How bioactive molecules are secreted from OSF in the OSCC microenvironment remains unclear.

The majority of cancer‐associated deaths and treatment failure amongst patients with cancer are not caused by the primary tumour but by distant metastasis, complex multistep processes including local invasion, proteolytic enzyme secretion, intravasation, extravasation and proliferation at distant sites.^14^ The epithelial‐mesenchymal transition (EMT) is a cellular process that transforms an epithelial cell into a mesenchymal phenotype and is essential for tumour migration, cancer stem cell properties, chemotherapeutic resistance and metastatic potential.^15^, ^16^ This process in pathological states results in the complete loss of epithelial cell‐cell junctions, such as α‐catenin, occludin, claudins and E‐cadherin, and the gain of mesenchymal phenotype markers, such as fibronectin, vimentin, N‐cadherin, Snail and Slug.^17^

Insulin‐like growth factor‐1 (IGF‐1) is a circulating polypeptide and the main potential mitogen for normal and neoplastic cells with a central role in cancer development and progression.^18^ IGF‐1 was shown to decrease programmed cell death and increase cancer cell differentiation.^19^ Increased IGF‐1 receptor (IGF‐1R) expression is associated with malignancy and tumour growth in different cancer types, including paraganglioma and oral carcinoma.^20^, ^21^ IGF‐1R expression is associated with poorer overall 5‐year survival in patients with stage III/IV OCSCC.^22^

IGF‐1 functions as a tumour promoter by increasing drug resistance^23^ and inducing EMT.^24^ In our previous study, the mRNA and protein expression levels of IGF‐1 were increased in human fibrotic buccal mucosal fibroblasts isolated from OSF specimens.^25^ Although OSF enhances oral cancer progression, the molecular events responsible for the microenvironment of oral cancer remain elusive. In the present work, we aimed to discuss the characteristics of the OSCC microenvironment and explore the contribution of OSF to EMT and OSCC tumour infiltration.

## 2 MATERIALS AND METHODS

### 2.1 Cell culture and ethics statement

Before entering this study, a written informed consent was obtained from each participant. Upon approval by the Institutional Review Board (IRB) of the Chung Shan Medical University Hospital (Taichung, Taiwan) on 27 February 2014 (IRB no. CS13250), normal human BMFs isolated from three normal controls and fBMFs from OSF tissues of three OSF patients were maintained in Dulbecco's modified Eagle's medium (DMEM) containing 10% v/v foetal bovine serum (FBS, Thermo Fisher Scientific Inc.) and 1% streptomycin/penicillin (Hyclone) and were characterized according to the morphology and OSF marker.

FaDu, a human epithelial cell line, derived from a squamous cell carcinoma of the hypopharynx tumour from a 56‐year‐old Hindu male patient, was acquired from American Type Culture Collection. OECM‐1, a human epithelial cell line, derived from a squamous cell carcinoma of the oral cavity from a Taiwanese male patient, was purchased from EMD Millipore Corporation. FaDu and OECM‐1 were grown in Roswell Park Memorial Institute (RPMI) 1640 medium with 10% v/v FBS and 1% streptomycin/penicillin. All cell cultures were maintained at 37°C incubation with 5% CO_2_.

### 2.2 Preparation of conditioned medium and treatment cells with CM

FBMFs (passages 3–10) were cultured in DMEM with 10% v/v FBS and 1% streptomycin/penicillin at a density of 2 × 10^5^ cells/10 cm dish until confluence for 5 days at 37°C incubation with 5% CO_2_, and then the CM was collected and centrifuged at 200 g for 5 min and frozen at −20°C until needed (not exceeding 2 weeks). For the treatment group, FaDu and OECM‐1 cells were grown in RPMI and CM at a ratio of 2:1. The medium was changed every 3 days. To exclude that the observed effects on OSCC cell invasion and migration were due to the amounts of serum in CM, FaDu and OECM‐1 cells were cultured in RPMI and DMEM standard culture media with 10% v/v FBS and 1% streptomycin/penicillin at a ratio of 2:1 in the control group.

### 2.3 Assessment of invasion by a Transwell

Transwell® polycarbonate membrane cell culture inserts (Corning, MA, USA) were coated with Matrigel® matrix (BD Biosciences) for the assessment of their invasive potential in vitro. After being pretreated with or without CM from fBMFs for 30 days, the FaDu and OECM‐1 cells were washed with phosphate buffer saline (PBS) and harvested and added to Transwell® inserts in serum‐free medium and then incubated for an additional 24 h at 37°C incubation with 5% CO_2_. The 8‐µm pore polycarbonate membrane inserts were coated with Corning® Matrigel® matrix (standard formulation with growth factor, product number: 354234, Corning Incorporated, Life Sciences), and the standard culture medium (RPMI with 10% v/v FBS) was added into the lower compartment of the apparatus. The invaded cells were fixed with 100% methanol for 20 min at room temperature and stained with Giemsa for 2 h. The cell numbers were photographed and counted under a light microscope. The data were expressed as the average cell numbers of five random fields for each treatment condition.

### 2.4 Wound healing migration assay

After being pretreated with or without CM from fBMFs for 30 days, the FaDu and OECM‐1 cells were plated onto six‐well plates until confluence for another 24 h. The FaDu cells were treated with or without 50 ng/ml IGF‐1 for 30 days, and the medium was changed every 3 days. NVP‐AEW541 (IGF‐IR inhibitor) was used for treatment or without for another 24 h, and then the cells were plated onto six‐well plates. The cells were wounded using culture inserts (Ibidi GmbH) to create a clear line and then incubated with RPMI containing 0.5% FBS for 12 (for FaDu) or 24 h (for OECM‐1). The cells that migrated into the wound area were photographed using a phase‐contrast microscope (×100).^26^

### 2.5 Determination of matrix metalloproteinase‐2 activity by gelatine zymography

The FaDu cells were treated with the CM of fBMF or 50 ng/ml IGF‐1 for 30 days, and the cells were washed with PBS and cultured in serum‐free RPMI medium for 24 or 48 h. The condition media (for 24 or 48 h) was collected and spun at 12,500 g for 5 min to remove any cell debris, and then the total number of viable FuDu cells was calculated using a Trypan Blue Exclusion Assay (Sigma Chemical Co.). The equal amounts of collected conditioned media (20 μl) were added with 5× non‐reducing sample buffer and loaded into 0.1% gelatin‒8% sodium dodecyl sulphate (SDS) polyacrylamide gel electrophoresis (PAGE) to examine the activity of MMP‐2. After electrophoresis, the gels were washed twice with wash buffer (2.5% Triton X‐100) and incubated in reaction buffer (40 mM Tris–HCl, pH 8.0, containing 0.02% NaN_3_ and 10 mM CaCl_2_) at 37°C for 12 h. The gel was then stained with Coomassie brilliant blue R‐250 for 30 min at room temperature and incubated with a destaining solution (10% methanol, 5% acetic acid in dH_2_O) until bands were visible.^27^ The stained gel was photographed and bands corresponding to the activity of MMP‐2 were quantified using ImageJ software (NIH). The quantification of the MMP‐2 activity was normalized by the total number of viable FuDu cells, calculated using a Trypan Blue Exclusion Assay.

### 2.6 Microculture tetrazolium proliferation assay

The FaDu and OECM‐1 cells were treated with or without CM (from fBMFs) for 30 days and then seeded in 24‐well plates at a density of 2 × 10^4^ cells/well for 24 h or 48 h. The cells were washed with PBS and incubated with 0.5 mg/ml 3‐(4,5‐dimethylthiazole‐2‐y1)‐2,5‐diphenyltetrazolium bromide (Sigma) in culture medium for another 4 h. The blue formazan crystals of viable cells were dissolved with 2‐propanol and measured by a Hitachi U‐1900 spectrophotometer (Hitachi) at 570 nm.^28^

### 2.7 Western blot analysis

The total cell lysates were collected using a cold mammalian protein extraction buffer kit (GE Healthcare Bio‐Sciences Corp.) containing protease inhibitor cocktails (Merck Millipore). The cell lysates were subjected to a centrifugation of 10,000 ***g*** for 30 min at 4°C, after a vortex at 0°C for 10 min. The concentration of the total protein was determined using the Bradford protein assay kit (Thermo Fisher Scientific). Equal amounts of extracted proteins (20 μg) were separated by 10% SDS‐PAGE and transferred onto a nitrocellulose membrane (GE Healthcare). The transferred membrane was incubated with MMP‐2 (cat. MAB3308; source: mouse; 1:1000 dilution; Sigma‐Aldrich; Merck KGaA), total focal adhesion kinase (FAK) (FAK, cat. #3285; source: rabbit), p‐FAK Tyr 925 (cat. #3284; source: rabbit), Ras‐related C3 botulinum toxin substrate 1 (Rac‐1)/cell division control protein 42 homologue (CDC42) (cat. #4651; source: rabbit), IGF‐1R (cat. #3027; source: rabbit), p‐IGF‐1R (cat. #3024; source: rabbit;), E‐cadherin (cat. #3195; source: rabbit), Ras homolog family member A (RhoA, cat. #2117; source: rabbit), Vimentin (cat. #3932; source: rabbit), N‐cadherin (cat. #4061; source: rabbit) (1:1000 dilution, Cell Singling Technology, Inc.), p‐FAK Tyr397 (cat. 611807; source: mouse) and Fibronectin (cat. 610078; source: mouse) (1:1000 dilution, BD Transduction Laboratories) antibodies, washed and monitored with an immunoblot assay using specific secondary antibodies. The bound primary antibodies were detected by a chemoluminescent reagent (Thermo Fisher Scientific), and the chemiluminescence signals were measured and quantified using a Luminescent Image Analyzer LAS‐4000 mini (GE Healthcare). After the intensity of each band was measured by densitometry, the relative intensities were calculated by normalizing to glyceraldehyde 3‐phosphate dehydrogenase (GAPDH, cat. sc‐32233; source: mouse) or β‐actin (cat. sc‐1615; source: goat) (1:1000 dilution; Santa Cruz Biotechnology, Inc.) from the corresponding sample.^29^

### 2.8 RNA preparation and TaqMan quantitative real‐time polymerase chain reaction

After the cells were pretreated with or without CM from fBMF cells for 30 days, the total cellular RNA was isolated from FaDu and OECM‐1 cells using Trizol reagent (Thermo Scientific, Waltham, MA, USA) in accordance with the manufacturer's instructions. Quantitative real‐time PCR analysis was conducted using TaqMan one‐step PCR Master Mix (Applied Biosystems). We added 100 ng of total cDNA per 25 μl reaction with MMP‐2 or GAPDH primers and TaqMan probes designed using commercial software. Quantitative real‐time PCR assays were performed in triplicate on a StepOnePlus sequence detection system. The threshold was set above the non‐template control background and within the linear phase of target gene amplification to calculate the cycle number at which the transcript was detected.

### 2.9 Immunofluorescence staining

The FaDu cells were treated with or without CM of fBMFs for 30 days or with or without IGF‐1 or NVP‐AEW541 (IGF‐1R inhibitor) for 30 days. The cells were then cultured on sterile glass coverslips in six‐well plates, washed in PBS for 20 min, fixed in 4% paraformaldehyde for 20 min, and permeabilized with 0.5% Triton X‐100 in PBS. The slides were incubated and stained by labelling F‐actin with Alexa Fluor®568 phalloidin (Thermofisher) overnight at 4°C. The nuclei were counterstained with 4'‐6‐diamidino‐2‐phenylindole (DAPI) and analysed by fluorescence microscopy.

### 2.10 Measurement of MMP‐2 promoter activity

The FaDu and OECM‐1 cells were treated with or without CM from fBMFs for 30 days. A 460 bp (−218 to +243) segment from the 5ʹ‐promoter region of the MMP‐2 gene was cloned. The pGL3‐MMP‐2 plasmids were transfected into FaDu or OECM‐1 cells using TurboFect transfection reagent (Thermo Scientific) in accordance with the manufacturer's instructions for 48 h. After transfection, the MMP‐2 promoter plasmids and cells lysates were prepared and examined by a luciferase assay system (Promega). Firefly luciferase activities were standardized for the β‐galactosidase activity.^30^

### 2.11 Cell‐matrix adhesion assay

The FaDu and OECM‐1 cells were treated with or without CM of fBMFs for 30 days. The cells were seeded into 24‐well dishes coated with collagen type I for 40 min. Non‐adherent cells were removed by washing the dishes with PBS. Adherent cells were fixed with 100% methanol for 20 min and stained with 0.1% crystal violet for 30 min. After staining, the fixed cells were lysed in 30% acetic acid in water, and the absorbance was measured at 550 nm with a Hitachi U‐1900 spectrophotometer (Hitachi).^27^

### 2.12 Human‐activated receptor tyrosine kinase array

The FaDu and OECM‐1 cells were treated with or without CM of fBMF cells for 30 days and then rinsed with PBS and solubilized cells at 1 × 10^7^ cells/ml in lysis buffer for 30 min. In brief, 300 μg of protein of whole‐cell lysates were incubated with human phospho‐RTK arrays (ARY001B) (R&D Systems), in which the arrays were incubated with the total cell lysates overnight at 4°C with shaking, washed with 1× washing buffer and incubated with 1X streptavidin‐horseradish peroxidase for 2 h at room temperature on a rocking platform shaker before being incubated with a Chemi Reagent Mix and exposure of the membranes to X‐ray film. The arrays were scanned with the Bio‐Rad Molecular Imager Gel Doc XR system.^27^

### 2.13 Animal study

The protocol was approved by the Institutional Animal Care and Use Committee (IACUC) of the Institutional Animal Welfare Guidelines of Chung Shan Medical University (IACUC Approval Number:1386). For the nude mouse xenograft model, immunodeficient male nude BALB/c AnN. Cg*Foxn ^nu^*/Crl Narl mice (age: 4–5 weeks old; weight: 17–19 g) were obtained from the National Laboratory Animal Center (Taipei, Taiwan). The drinking water and food of the mice were treated with sterility and maintained in a pathogen‐free environment at the Laboratory Animal Center of Chung Shan Medical University. OECM‐1 cells were transfected with the pGL4.50[luc2/CMV/Hygro] vector encoding the luciferase reporter gene luc2 (originating from the North American firefly, Photinus pyralis). The stable luciferase‐expressing OECM‐1 cells were selected by hygromycin B (400 μg/ml) in growth medium. Luciferase‐expressing OECM‐1 cells (5 × 10^6^ cells/0.1 ml/mouse) were treated with or without the CM of fBMF cells for 30 days and injected subcutaneously into the upper right and left front axilla region of the nude mice (*n* = 5 for each group). After 43 days, each mouse was intraperitoneally injected with 150 mg D‐luciferin/kg bodyweight and was anaesthetized with inhaled 2% isoflurane. Tumour growth was performed using an IVIS 50 animal imaging system (Xenogen Corporation), and the signal intensity was analysed using live imaging software.^27^ After 45 days, the animals were euthanized with 30–70% displacement rate of chamber air with CO_2_ gas/min.

### 2.14 Bio‐Plex Pro‐human cytokine assay

FBMFs were cultured in DMEM with 10% FBS and 1% streptomycin/penicillin at a density of 2 × 10^5^ cells/10 cm dish until confluence for 5 days at 37°C incubation with 5% CO_2_, and then the CM was collected and centrifuged at 1000 rpm for 5 min and frozen at −20°C until needed (not exceeding 1 week). For the control group, DMEM with 10% FBS was used. The cytokine profiles in the CM of fBMF cells were analysed using a Bio‐Plex Pro‐human cytokine 27‐plex assay (Bio‐Rad Laboratories) in accordance with the manufacturer's instructions. In brief, 100 μl of assay buffer and 50 μL of beads were added to the assay plate. The standard (27 types of cytokines) was reconstituted and diluted in a fourfold dilution series. The samples (50 μl) were added to each well and rinsed twice with wash buffer. After 1 h of shaking in the dark, the wells were rinsed three times with wash buffer before the detection antibody was added to each well. After another washing step, 50 μl of streptavidin‐phycoerythrin solution was added to each well, and they were incubated for 10 min. After the last incubation step, the beads were resuspended in 125 μl of assay buffer with shaking at 1100 rpm for 30 s. The cytokine data were analysed using the Bio‐Plex 200 suspension array system (Bio‐Rad Laboratories). The cytokine protein expression of fBMF was compared with the standard DMEM with 10% FBS (control group).

### 2.15 Colony formation assay

The FaDu and OECM‐1 cells were treated with or without CM from fBMFs for 30 days, and then five thousand OECM‐1 or FaDu cells were plated as single cells in a six‐well plate for 8 days. The medium was changed every 3 days. After fixing with methanol, the cells were stained with crystal violet (Millipore, Sigma) for 30 min.

### 2.16 IGF‐1R expression analysis of The Cancer Genome Atlas

The Cancer Genome Atlas, a landmark cancer genomics programme, was used to dissect the clinical significance of IGF‐1R in the oral squamous cell carcinoma data set. The correlations amongst the mRNA levels of IGF‐1R and MMP‐2 in OSCC (*n* = 328) were examined. The correlation coefficient *r* measured the strength and direction of a linear relationship between IGF‐1R and MMP‐2 on a scatterplot. Box plots for the IGF‐1R expression values were generated with respect to the overall survival using the Kaplan‐Meier method. The *P*‐values were determined using a log‐rank test.^31^

### 2.17 Statistical analysis

The statistical analysis was conducted using one‐way analysis of variance (ANOVA) with a post hoc Dunnett's test (GraphPad Software). The *p*‐values less than 0.05 were considered as statistically significant differences.

## 3 RESULTS

### 3.1 OSF induces the invasion and migration of OSCC

As OSF is a chronic fibrosis with a long‐term effect in the tumour microenvironment, in this study, oral cancer cell lines were treated with CM of fBMF for an extended duration (30 days) to simulate OSF on the oral cancer process and induce the invasion and EMT of OSCC, verifying the importance of OSF for the carcinogenesis of OSCC. The expression and activity of proteinases from cancer cells elevate their invasion and metastasis, and cancer develops in a complex process and long‐term progression. We investigated the long‐term effects (30 days) of OSF on oral cancer cells.

**FIGURE 1:**
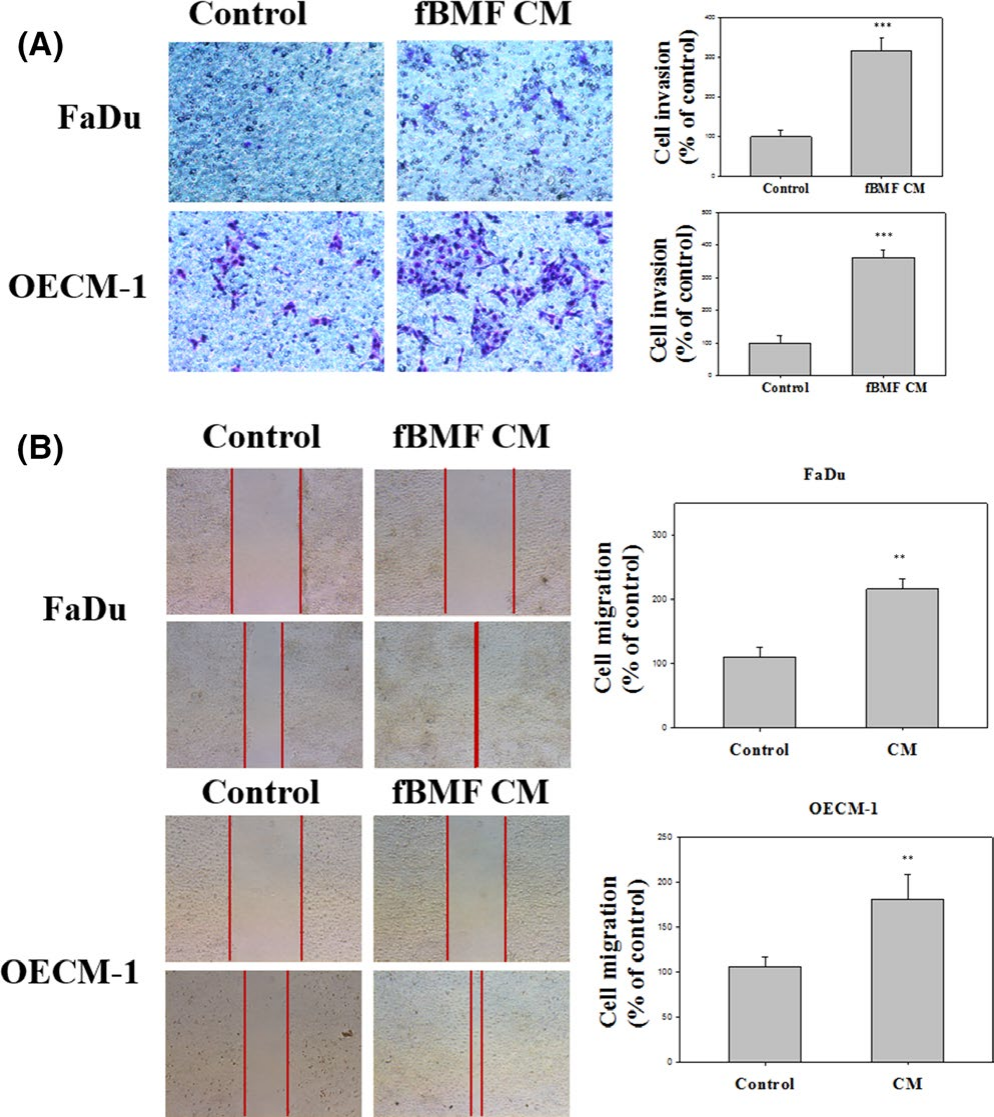
OSF increased the invasion and migration of FaDu and OECM‐1 cells. (A) FaDu and OECM‐1 cells were treated with the CM of fBMF for 30 days with a cell invasion assay (100×). (B) FaDu and OECM‐1 cells were treated with the CM of fBMF by wound‐healing assay (40×). Quantitative data are presented as the means ± SD of three independent experiments (**, *p* < 0.01; ***, *p* < 0.001). The results from three repeated and separated experiments were similar

FBMFs were established from patients with OSF, and the FaDu and OECM‐1 cells were treated with the CM of fBMFs for 30 days by Transwell invasion assay and a wound healing migration assay to determine whether fBMFs can affect the invasion and migration of OSCC. After treatment with the CM of fBMFs, cell invasion was increased in FaDu and OECM‐1 cells. Quantitative analysis revealed that the invasion activities of FaDu and OECM‐1 cells were increased by 316.0% (*p* < 0.001) and 362.7% (*p* < 0.001), when the cells were treated with fBMFs (Figure 1A).

The wound healing migration assay showed that fBMFs significantly increased the migration capability of FaDu and OECM‐1 cells (Figure 1B). These data indicated that the invasion and migration activity of FaDu and OECM‐1 treated with fBMFs were increased for a long time treatment.

### 3.2 OSF exerts an increased effect on MMP‐2 of OSCC

**FIGURE 2:**
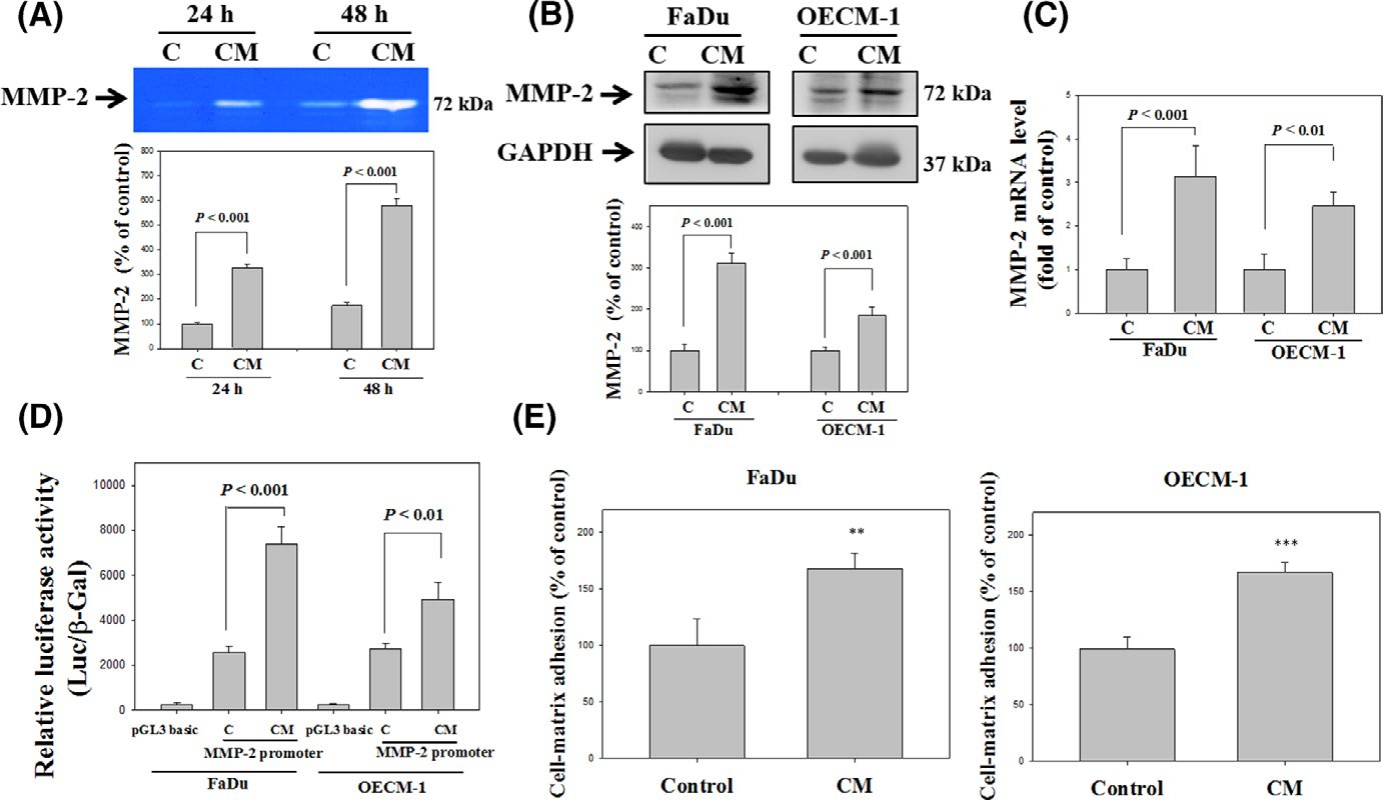
Effects of OSF on MMP‐2 and cell‐matrix adhesion of oral cancer cells. (A) FaDu cells were treated with the CM of fBMF for 30 days, and then condition media (for 24 and 48 h) was collected for the analysis of MMP‐2 with gelatine zymography. (B) Western blot analysis of the protein expression of MMP‐2 with GAPDH as an internal control in FaDu and OECM‐1 cells after 30 days of treatment with the CM of fBMF. (C) The MMP‐2 mRNA expression of FaDu and OECM‐1 after 30 days of treatment with CM of fBMF by real‐time PCR. (D) The luciferase activity was measured in transiently transfected FaDu and OECM‐1 cells using pGL3‐MMP‐2 after 30 days of treatment with CM of fBMF. (E) The cell‐matrix adhesion was measured in FaDu and OECM‐1 cells after 30 days of treatment with CM of fBMF. The results from three repeated and separated experiments were similar. Quantitative data are presented as the means ± SD of three independent experiments. The statistical significance was analysed through one‐way ANOVA with a post hoc Dunnett's test (*, *p* < 0.05; **, *p* < 0.01; and ***, *p* < 0.001)

The FaDu cells were treated with the CM of fBMFs for 30 days, and then the conditioned media (for 24 and 48 h) of the FaDu cells was collected for the analysis of MMP‐2 activity by gelatine zymography. The data indicated that fBMFs significantly elevated the activity of MMP‐2 in FaDu cells (Figure 2A). The MMP‐2 protein level was also examined using a western blot assay, and the expression of MMP‐2 of the FaDu and OECM‐1 cells was increased with fBMF treatment (Figure 2B). The mRNA expression (Figure 2C) and MMP‐2 transcription activity (Figure 2D) of FaDu and OECM‐1 cells increased when treated with fBMFs. fBMF treatment also elevated the cell‐matrix interactions of FaDu and OECM‐1 cells (Figure 2E).

### 3.3 OSF reduces the cell proliferation and tumour growth of OSCC

**FIGURE 3:**
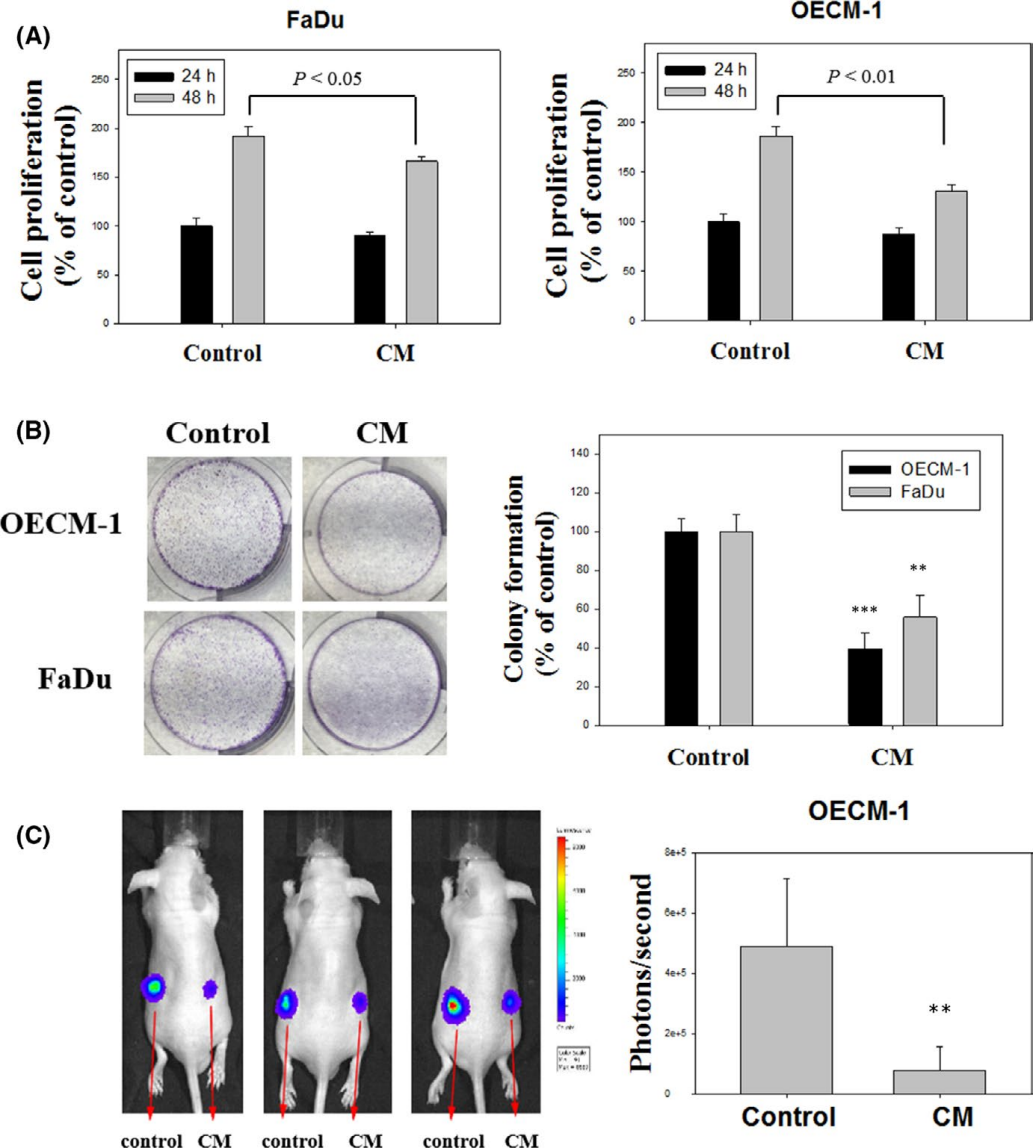
Effects of OSF on the cell growth of oral cancer cells. (A) FaDu and OECM‐1 cells were treated with the CM of fBMF for 30 days, and the cell proliferation was determined with a microculture MTT assay for 24 and 48 h. The results from three repeated and separated experiments were similar. Quantitative data are presented as the means ± SD of three independent experiments. (B) Cells were plated as single cells for 8 days in a 6‐well plate, and then the number of formed cell colonies were determined. (C) Bioluminescence over time after the subcutaneous implantation of luciferase‐expressing OECM‐1 cells

The FaDu and OECM‐1 cells were cultured with or without CM from fBMFs for 30 days, and the cell proliferation of oral cancer cells was investigated using an MTT assay. OSF inhibited the cell growth of FaDu and OECM‐1 cells compared with the 48 h control group (Figure 3A). We performed a colony formation assay to examine the long‐term effect of fBMF‐reduced cell growth. The colonies number of OECM‐1 and FaDu cells significantly reduced after fBMFs CM treatment (Figure 3B). Luciferase‐expressing OECM‐1 cells were treated with or without CM from fBMFs for 30 days and then subcutaneously inoculated into the right and left flank of immunodeficient nude mice, respectively, to determine the in vivo effects of OSF. The tumour growth of the fBMF‐treated groups was remarkably inhibited compared with that of the control groups (Figure 3C).

### 3.4 OSF improved the EMT of OSCC

**FIGURE 4:**
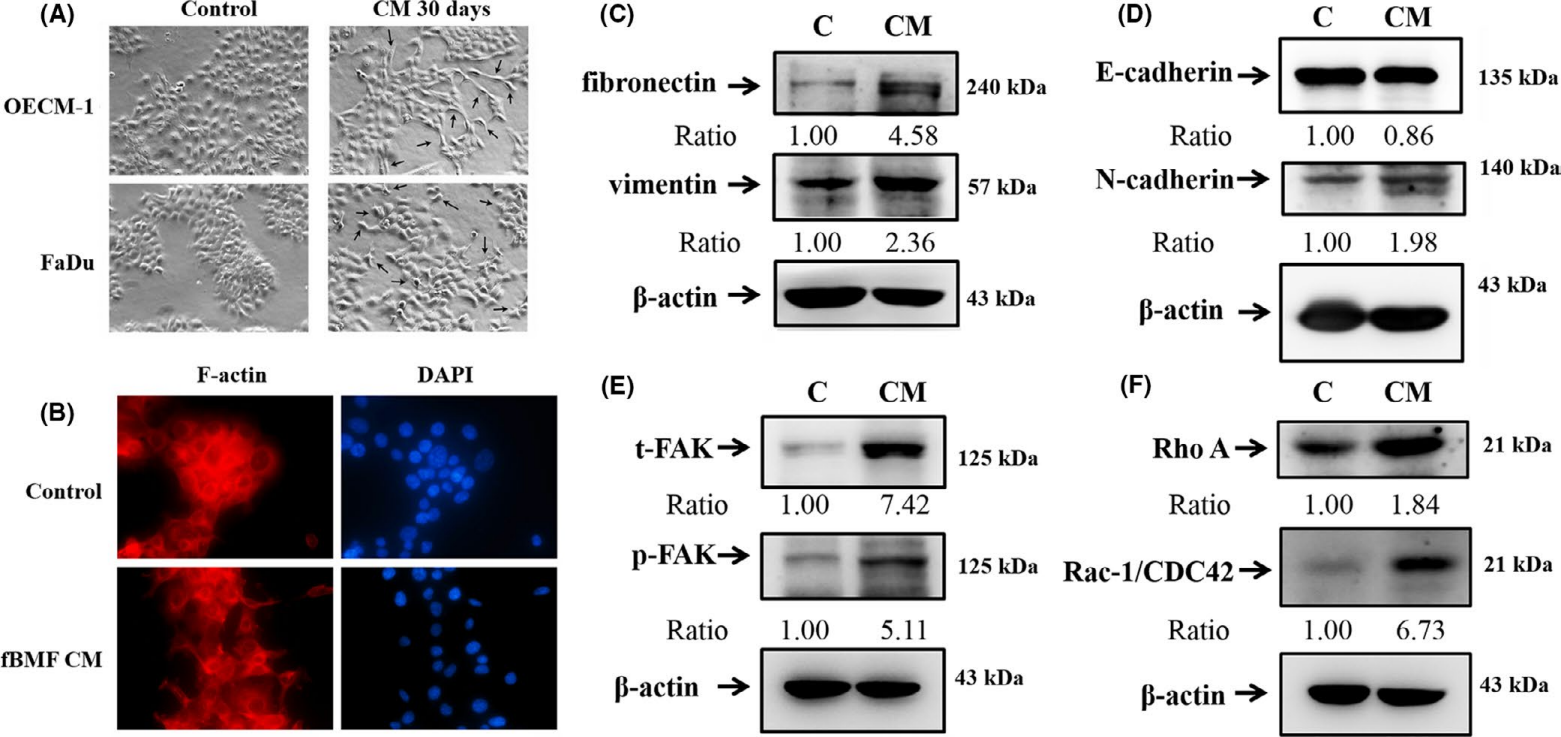
Effects of CM of fBMF on the morphology and the cytoskeleton related protein in human oral cancer cells. OECM‐1 and FaDu cells were treated with the CM of fBMF for 30 days. (A) Phase image of cells (100×). (B) Immunofluorescence staining of FaDu cells (treated with CM of OSF for 30 days) with Texas‐568 phalloidin to visualize the actin cytoskeleton. The nuclei were labelled using DAPI stain. The results from three repeated and separated experiments were similar. The cell lysates were then subjected to western blot analysis. (C) Fibronectin and vimentin with β‐actin as an internal control. (D) E‐cadherin and N‐cadherin with β‐actin as an internal control. (E) t‐FAK and p‐FAK, (F) Rho A and Rac‐1/CDC42 with β‐actin as an internal control. The results from three repeated and separated experiments were similar

OECM‐1 and FaDu cells were pretreated with or without the CM of fBMFs for 30 days to elucidate whether the OSF could affect cell morphology changes. The cells exhibited fibroblast‐like morphology and suppressed cell‐to‐cell contact (Figure 4A). The CM of fBMFs induced cell scattering and fibroblast‐like morphology as investigated by immunofluorescence staining with Texas‐568 phalloidin to visualize the actin cytoskeleton (Figure 4B). The effect of OSF on the major regulators and markers of EMT in OSCC was also analysed. The CM of fBMFs significantly increased the protein expression levels of fibronectin, vimentin (Figure 4C), N‐cadherin (Figure 4D), FAK, p‐FAK (Figure 4E), Rac‐1/CDC42 and RhoA (Figure 4F) but downregulated the E‐cadherin expression in FaDu cells (Figure 4D).

### 3.5 OSF promoted EMT and invasion by upregulating IGF‐1R expression in human oral cancer

**FIGURE 5:**
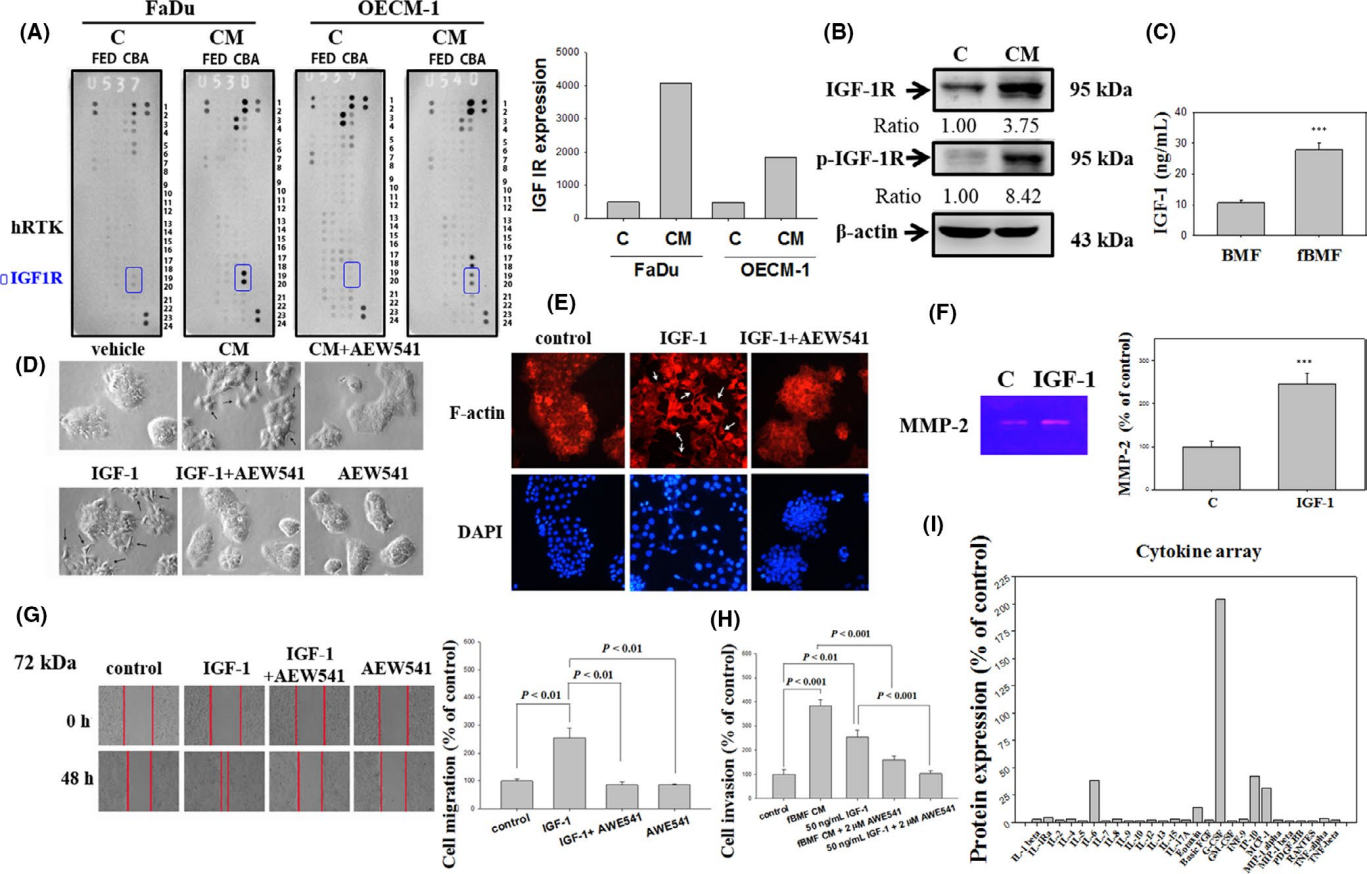
Effects of IGF‐1 on the morphology, MMP‐2, and invasion in oral cancer cells. (A) FaDu and OECM‐1 cells were treated with the CM of fBMF for 30 days, and cell lysates were used to detect 49 different receptor tyrosine kinases by human phosphor‐RTK array (from R&D system). (B) FaDu cells were treated with the CM of fBMF for 30 days, and the cell lysates were subjected to western blot analysis to detect IGF‐1 receptor (IGF‐1R) and p‐IGF‐1R with β‐actin as an internal control. (C) CM was collected from fBMFs and analysed for IGF‐1 with an ELISA kit. (D) FaDu cells were treated with the CM of fBMF and 50 ng IGF‐1 with or without 2 μM NVP‐AEW541. The cells were imaged with a microscope (100×). (E) Immunofluorescence staining of FaDu cells (treated with or without IGF‐1 or NVP‐AEW541 for 30 days) with Texas‐568 phalloidin was used to visualize the actin cytoskeleton (400×). (F) FaDu cells were treated with 50 ng/ml IGF‐1 for 30 days, and then the condition media (for 24 h) was collected for the analysis of MMP‐2 by gelatine zymography. (G) FaDu cells were treated with IGF‐1 with or without NVP‐AEW541 by a wound healing assay. (H) FaDu cells were treated with CM of fBMF and 50 ng/ml IGF‐1 with or without 2 μM NVP‐AEW541 by a cell invasion assay (40×). (I) Cytokines from fBMFs were analysed using a Bio‐Plex Pro‐human cytokine 27‐plex assay. CM was collected from fBMFs and was analysed for 27 different cytokines. The cytokine expression of fBMFs was compared with standard DMEM with 10% FBS as a control group. Results representing three separate experiments are shown for B–H, and the statistical significance was analysed through one‐way ANOVA with a post hoc Dunnett's test (***, *p* < 0.001)

Considering that OSF significantly elevates the invasion and migration of cancer cells, we subsequently examined whether OSF influenced their receptor tyrosine kinase (RTK). A human‐activated RTK array was used to screen for the expression levels of several RTK following the treatment of FaDu and OECM‐1 cells with fBMF CM for 30 days. The expression levels of IGF‐1R increased after fBMF CM treatment in FaDu and OECM‐1 cells (Figure 5A). After the FaDu cells were treated with fBMF CM for 30 days, the protein expression levels of IGF‐1R and phosphorylated IGF‐1R significantly increased as determined by western blot analysis (Figure 5B). The IGF‐1 expression of fBMFs was also investigated through an IGF‐1 ELISA assay kit to clarify the involvement of IGF‐1 in increasing the invasion by fBMF CM treatment.

The results revealed that IGF‐1 expression was increased in OSF region compared with the BMF groups (Figure 5C). The FaDu cells were treated with NVP‐AEW541 (IGF‐IR inhibitor) to disrupt the IGF‐1 signalling to examine the role of IGF‐1 in inducing EMT and invasion in oral cancer cells. IGF‐1R inhibition by the potent inhibitor NVP‐AEW541 reversed the OSF‐induced morphology change, and the combined treatment of IGF‐1 and NVP‐AEW541 showed an inhibitory effect on IGF‐1‐induced mesenchymal transformation into an epithelial phenotype (Figure 5D). After FaDu cells were treated with IGF‐1, the cells showed a fibroblast‐like appearance and exhibited lost cell‐to‐cell contact. NVP‐AEW541 reversed IGF‐1‐induced scattering, as determined by immunofluorescence staining with Texas‐568 phalloidin to visualize the actin filaments (Figure 5E).

A gelatine zymography assay revealed that IGF‐1 elevated the MMP‐2 activity of FaDu cells (Figure 5F). After treatment with IGF‐1, the migration and invasion of the FaDu cells were increased, and NVP‐AEW541 attenuated the IGF‐1‐induced migration (Figure 5G) and invasion (Figure 5H). Thus, IGF‐1 was elevated in OSF and, consequently, increased the expression of IGF‐IR and promoted the EMT and invasion by upregulating IGF‐1R in human oral cancer.

A Bio‐Plex Pro‐human cytokine 27‐plex assay was employed to identify the secretion of bioactive molecules from OSF in an OSCC microenvironment. The results revealed that the interleukin‐6 (IL‐6), eotaxin, granulocyte colony‐stimulating factor (G‐CSF), interferon gamma‐induced protein 10 (IP‐10), and monocyte chemoattractant protein‐1 (MCP‐1) secretions were increased by fBMFs (Figure 5I).

### 3.6 IGF‐1R is increased in patients with OSCC

**FIGURE 6:**
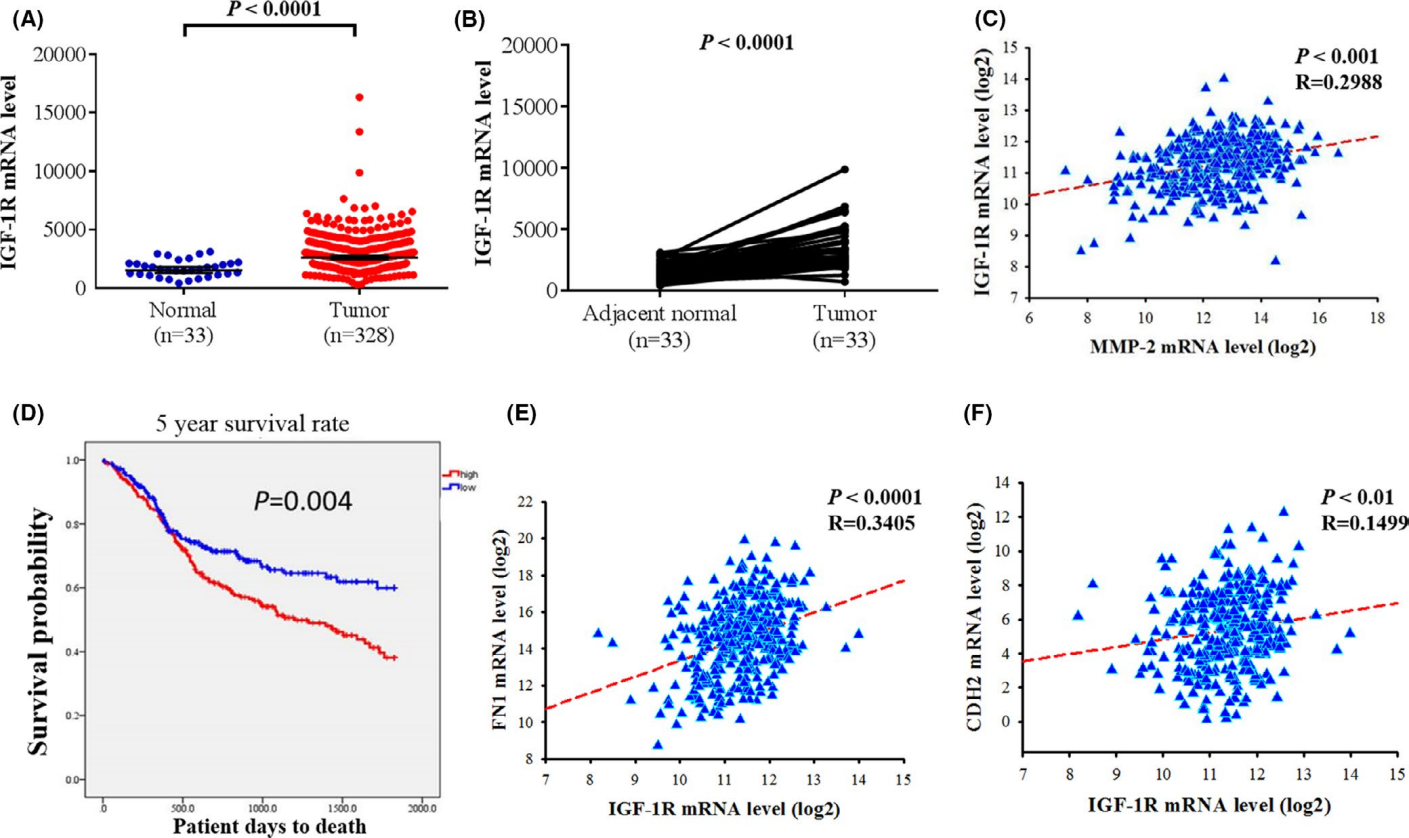
Levels of IGF‐1R were increased in oral squamous cell carcinoma samples. (A) The IGF‐1R expression in normal and oral squamous cell carcinoma from The Cancer Genome Atlas (TCGA) Data Portal. (B) The relative expression of IGF‐1R in 33 pairs of oral squamous cell carcinoma tumour tissues and their corresponding adjacent non‐cancerous tissues. (C) Correlations between the mRNA levels of MMP‐2 and IGF‐1R in oral squamous cell carcinoma. A significant correlation was found between MMP‐2 and IGF‐1R (Spearman rank correlation coefficient *r* = 0.2988, *p* < 0.0001). (D) 5‐year survival curve for patients with high (red lines) and low (blue lines) IGF‐1R mRNA expression levels using the Kaplan–Meier method. (E) Correlations between the mRNA levels of fibronectin (FN1) and IGF‐1R in oral squamous cell carcinoma. A significant correlation was found between FN1 and IGF‐1R (Spearman rank correlation coefficient *r* = 0.3405, *p* < 0.0001). (F) Correlations between the mRNA levels of Cadherin‐2 (CDH2) and IGF‐1R in oral squamous cell carcinoma. A significant correlation was found between CDH2 and IGF‐1R (Spearman rank correlation coefficient *r* = 0.1499, *p* < 0.01). The *p* values were determined using a log‐rank test

The Cancer Genome Atlas (TCGA) OSCC data set (*n* = 328) was further used to verify our findings. The mRNA expression of IGF‐1R in OSCC tissues was higher than that in normal tissues (*p* < 0.0001; Figure 6A). Higher IGF‐1R levels were detected in OSCC tissues (n = 33) compared with their corresponding adjacent non‐cancerous tissues (*p* < 0.0001; Figure 6B). A positive correlation was found between the MMP‐2 and IGF‐1R expression in OSCC tumours (*p* < 0.0001; Figure 6C). The results from the TCGA data set of patients with OSCC indicated that high IGF‐1R expression was associated with a poor survival rate (*p* = 0.004; Figure 6D).

Next, we evaluated the correlation between the expression of EMT marker and IGF‐1R. A positive correlation was found between the fibronectin (FN1) and IGF‐1R expression in OSCC tumours (*p* < 0.0001; Figure 6E). A positive correlation was also found between Cadherin‐2 (CDH2, also known as N‐cadherin) and IGF‐1R expression in OSCC tumours (*p* < 0.01; Figure 6F). These data suggest that IGF‐1R expression may promote the mortality of patients with OSCC.

**FIGURE 7:**
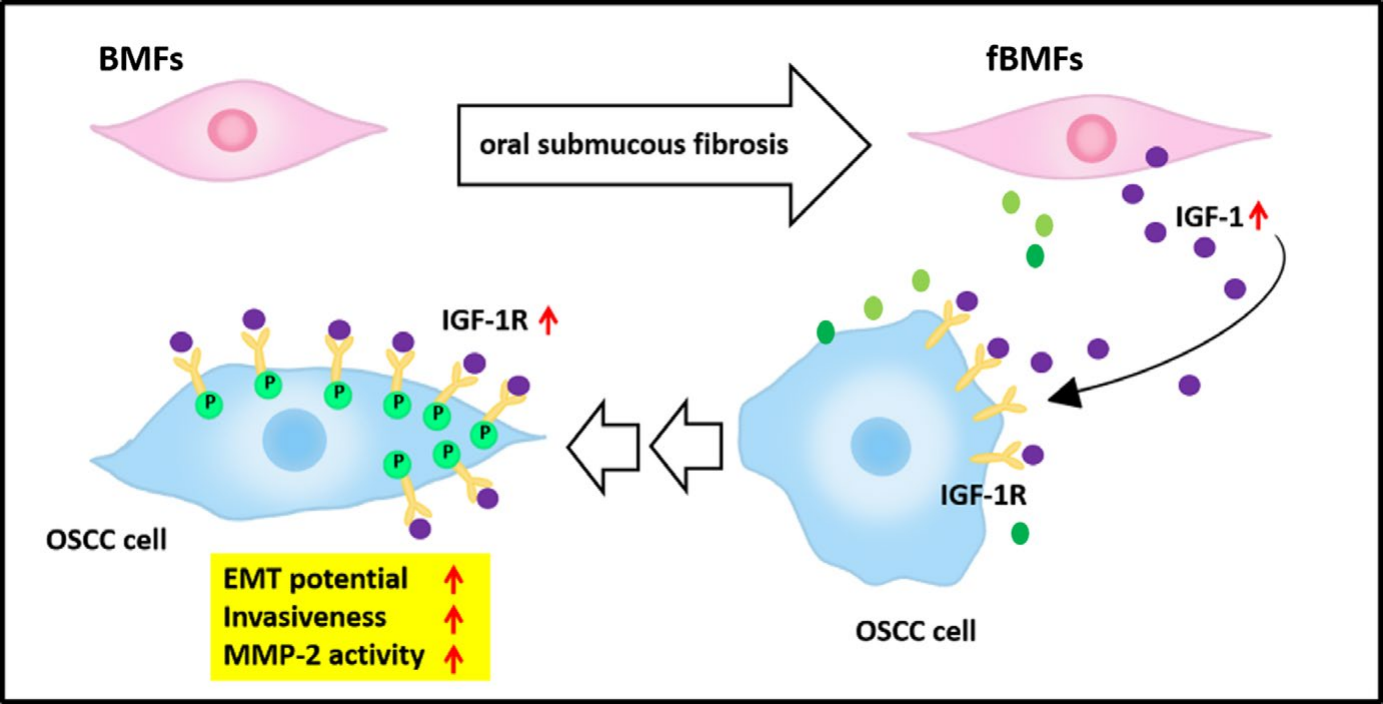
Tumour‐promoting activity of OSF on human OSCC cells is attributed to the activation of IGF‐1R, invasion and EMT in human OSCC cells

Taken together, we provided molecular evidence of the tumour‐promoting activity of OSF‐derived fibroblast on OSCC cells by showing marked induction of the IGF‐1 production. These findings suggested that OSF‐derived fibroblast is able to promote migration and invasion and induce an epithelial‐mesenchymal‐like state via the upregulation of IGF‐1R in oral cancer cells (Figure 7).

## 4 DISCUSSION

EMT activation plays an essential role in the initial oral cancer metastasis and generates cancer stem cells in OSCC.^16^, ^32^ During EMT, epithelial cells change their adherent epithelial morphology to the fibroblast‐like and maintenance migratory phenotypes in cancer cells. Cell‐cell adhesion between epithelial cells is disrupted by downregulating epithelial markers, such as E‐cadherin, α‐catenin, occludins and claudins. The cells undergoing EMT aberrantly express high levels of mesenchymal markers, such as ZEB, N‐cadherin, vimentin and fibronectin^33^, ^34^ as well as MMP secretions.^35^

MMP‐2 (gelatinase A), which is a type IV collagenase, is involved in the remodelling and degradation of the extracellular matrix and plays a central role in the tumour metastasis, invasion and shortened survival of patients with cancer.^36^ EMT markers, which are mediators of EMT regulated complex signalling pathways have detrimental role in the pathogenesis of OSF and OSCC.^37^ IGF‐I/IGF‐IR signalling is associated EMT progression in various type of cancers.^38^ Our data indicated that OSF remarkably increased the cell‐matrix adhesion, invasion and migration abilities and the activity of MMP‐2 of oral cancer and affected the EMT by enhancing the expression of N‐cadherin, fibronectin and vimentin and downregulating the expression of E‐cadherin in human oral cancer cells.

EMT is induced by several signalling pathways, including Hedgehog, Wnt/β‐catenin, hypoxia and TGF‐β1. TGF‐β1 is a multifunctional growth factor and plays a dual role in pro‐ and anti‐tumoral effects. The expression of TGF‐β1 was correlated with the induction of the angiogenic pathway in OSCC.^39^ TGF‐β1 is also a potent inhibitor of epithelial cell growth in the early stages of carcinogenesis in cancer, and this property contributes to its role as a tumour‐suppressor.^40^ Earlier reports indicated that high expression EMT markers in malignant mesenchymal tumours will reduce tumour growth. Researchers suggested that turning off EMT‐inducing transcription factor Twist1 expression to allow reversion of the EMT is essential for disseminated tumour cells to proliferate and promotes colonization in distant sites.^41^ The data presented here demonstrate that OSF induced the invasion and EMT of FaDu and OECM‐1 cells but reduced the cell proliferation and tumour growth in vivo.

IGF‐1 plays an essential role in cell growth and cytoskeletal rearrangements.^42^ After the IGF‐1 receptor binds to IGF‐1, the autophosphorylation of this receptor at its tyrosine residues can lead to the formation of focal adhesions, in which FAK is recruited rapidly and phosphorylated, thus, activating the downstream molecules in triple‐negative breast cancer.^43^ FAK phosphorylation initiates a cascade of events that are involved in the progression of malignancies, such as proliferation, migration, invasion, angiogenesis and metastasis.^44^ FAK signalling regulates MMP‐2 expression and the migration and invasion of human glioblastomas.^45^

IGF‐1activates the Rho/Rho‐kinase pathway and elevates the formation of stress fibres by inhibiting the ectopic expression of the postsynaptic density protein 95/ discs‐large / zona occludens 1 and regulator of G protein signalling domains of leukaemia‐associated Rho guanine nucleotide exchange factor.^42^ RhoA mediates the cell motility of many diverse types of cancer as indicated by its regulation of cytoskeletal organization and gene expression.^46^ RhoA is also implicated in mediating the activation of FAK in response to the development of aggressive non‐small cell lung cancer.^47^ Our results showed that OSF activated IGF‐1R and increased RhoA expression and FAK activation in human OSCC. This phenomenon indicates that IGF‐1 from OSF has a potential role in increasing cancer progression in the OSCC microenvironment.

IGF‐1 is a 70‐amino acid polypeptide hormone and a major determinant in cancer development and pathogenesis.^42^ The insulin/IGF system was implicated in the development of drug resistance to epidermal growth factor receptor‐targeted agents and chemotherapeutic drugs in colorectal cancer.^48^ Increased IGF‐1R signalling is associated with a poor response to anti‐EGFR treatment in head and neck cancer cells.^49^ IGF‐1 promotes the motility of muller glial cells by activating MMP‐2 and the phosphoinositide 3‐kinase signalling pathway.^50^

High IGF‐1R expression in tumour cells is associated with poor outcomes in patients with resected pancreatic ductal adenocarcinoma.^51^ In this work, patients with OSCC and high IGFR expression levels had poorer 5‐year survival than those with low IGFR expression according to the TCGA patient transcriptome data. A positive correlation was found between the MMP‐2 and IGF‐1R expression in OSCC tumours.

CAFs, which are frequently present in human carcinomas, have emerged as a prominent modifier of cancer progression, and they produce inflammatory cytokines, chemokines and numerous growth factors, and these secretory products facilitate communication between CAFs and cancer cells, resulting in cancer progression.^52^ In the current study, five cytokines, including IL‐6, eotaxin, G‐CSF, IP‐10 and MCP‐1 were increased by fBMFs in the Bio‐Plex Pro‐human cytokine assay. These five cytokines may require penetration to induce cell invasion and migration. Further research is required to verify the role of cytokines and factor expression by fBMFs in the progression of OSCC.

A positive correlation was also found between EMT markers (FN1 and CDH2) and the IGF‐1R expression in OSCC tumours. OSF was reported to be associated with better prognosis amongst OSCC patients.^53^ As the TCGA data did not provide the cases that may have fibrosis in OSCC patients, there was a limitation in linking oral fibrosis and IGF‐1R expression.

The tumour microenvironment is considered as an attractive target for new anti‐tumour therapies for patients with OSCC. OSF and cancer‐associated fibroblasts have been shown to have tumour‐promoting effects in oral cancer. This study discusses the characteristics of the microenvironment of OSCC and investigates the contribution of OSF to the epithelial‐mesenchymal transition and tumour infiltration of OSCC. The present study showed that the tumour‐promoting activity of OSF on human OSCC cells was attributed to the activation of IGF‐1R, invasion, and EMT in human OSCC cells. Additionally, the high IGF‐1R expression was associated with a poor survival rate in patients with OSCC. These data suggest that IGF‐1R may promote the mortality of patients with OSCC. IGF‐1, which is secreted in OSF, promoted the migration ability in oral cancer cells.

## CONFLICTS OF INTEREST

The authors declare that there is no conflict of interest.

## AUTHOR CONTRIBUTION

**Pei‐Ni Chen:** Investigation (lead); Resources (equal); Writing‐original draft (equal); Writing‐review & editing (equal). **Chiao‐Wen Lin:** Conceptualization (equal); Investigation (supporting); Methodology (equal); Writing‐review & editing (equal). **Shun‐Fa Yang:** Conceptualization (equal); Investigation (supporting); Methodology (equal). **Yu‐Chao Chang:** Conceptualization (equal); Methodology (equal); Writing‐original draft (equal); Writing‐review & editing (equal).

## Data Availability

The data used to support the findings of the present study are available from the corresponding author upon request.
